# Advancing human health in the decade ahead: pregnancy as a key window for discovery: A Burroughs Wellcome Fund Pregnancy Think Tank

**DOI:** 10.1016/j.ajog.2020.06.031

**Published:** 2020-09

**Authors:** Yoel Sadovsky, Sam Mesiano, Graham J. Burton, Michelle Lampl, Jeffrey C. Murray, Rachel M. Freathy, Anita Mahadevan-Jansen, Ashley Moffett, Nathan D. Price, Paul H. Wise, Derek E. Wildman, Ralph Snyderman, Nigel Paneth, John Anthony Capra, Marcelo A. Nobrega, Yaacov Barak, Louis J. Muglia, Burroughs Wellcome Fund Pregnancy Think Tank Working Group

**Affiliations:** a Department of Obstetrics, Gynecology, and Reproductive Sciences, Magee-Womens Research Institute, University of Pittsburgh, Pittsburgh, PA; b Department of Reproductive Biology, Case Western Reserve University, and Department of Obstetrics and Gynecology, University Hospitals of Cleveland, Cleveland, OH; c Department of Pathology; d Centre for Trophoblast Research; e University of Cambridge, Cambridge, United Kingdom; f Center for the Study of Human Health, Emory University, Atlanta, GA; g Department of Pediatrics, University of Iowa, Iowa City, IA; h University of Exeter, Exeter, United Kingdom; i Vanderbilt Biophotonics Center and Department of Biomedical Engineering, Vanderbilt University, Nashville, TN; j Department of Biological Sciences, Vanderbilt University, Nashville, TN; k Institute for Systems Biology, Seattle, WA; l Department of Pediatrics, Stanford University School of Medicine, Stanford University, Stanford, CA; m Genomics Program, College of Public Health, University of South Florida, Tampa, FL; n Duke Center for Personalized Health Care, Duke University Medical Center, Durham, NC; o Departments of Epidemiology and Biostatistics and of Pediatrics and Human Development, College of Human Medicine, Michigan State University, East Lansing, MI; p Department of Human Genetics, University of Chicago, Chicago, IL; q Office of the President, Burroughs Wellcome Fund, Research Triangle Park, NC

**Keywords:** adverse outcomes, artificial intelligence, birthweight, computational biology, developmental origins of adult disease, disparities, drug discovery, evolutionary biology, fetal growth, fetal growth restriction, genomics, gestational diabetes mellitus, immune tolerance, maternal health, metabolomics, multiomics, parturition, physiology, population health, precision medicine, preeclampsia, pregnancy, preterm birth, stillbirth

## Abstract

Recent revolutionary advances at the intersection of medicine, omics, data sciences, computing, epidemiology, and related technologies inspire us to ponder their impact on health. Their potential impact is particularly germane to the biology of pregnancy and perinatal medicine, where limited improvement in health outcomes for women and children has remained a global challenge. We assembled a group of experts to establish a Pregnancy Think Tank to discuss a broad spectrum of major gestational disorders and adverse pregnancy outcomes that affect maternal-infant lifelong health and should serve as targets for leveraging the many recent advances. This report reflects avenues for future effects that hold great potential in 3 major areas: developmental genomics, including the application of methodologies designed to bridge genotypes, physiology, and diseases, addressing vexing questions in early human development; gestational physiology, from immune tolerance to growth and the timing of parturition; and personalized and population medicine, focusing on amalgamating health record data and deep phenotypes to create broad knowledge that can be integrated into healthcare systems and drive discovery to address pregnancy-related disease and promote general health. We propose a series of questions reflecting development, systems biology, diseases, clinical approaches and tools, and population health, and a call for scientific action. Clearly, transdisciplinary science must advance and accelerate to address adverse pregnancy outcomes. Disciplines not traditionally involved in the reproductive sciences, such as computer science, engineering, mathematics, and pharmacology, should be engaged at the study design phase to optimize the information gathered and to identify and further evaluate potentially actionable therapeutic targets. Information sources should include noninvasive personalized sensors and monitors, alongside instructive “liquid biopsies” for noninvasive pregnancy assessment. Future research should also address the diversity of human cohorts in terms of geography, racial and ethnic distributions, and social and health disparities. Modern technologies, for both data-gathering and data-analyzing, make this possible at a scale that was previously unachievable. Finally, the psychosocial and economic environment in which pregnancy takes place must be considered to promote the health and wellness of communities worldwide.

Major adverse pregnancy-specific complications—preterm birth, preeclampsia, fetal growth restriction, and stillbirth—remain leading causes of maternal, fetal, and neonatal morbidity and mortality and are associated with increased disease risk across the lifespan.^1^ Despite growing attention, much greater progress is needed in revealing mechanisms and pathogenic pathways, advancing clinical diagnostic and therapeutic tools, and designing preventive strategies to push the field forward into the 22nd century. Modern technologies such as next-generation sequencing, artificial intelligence, machine learning, electronic medical records, and noninvasive imaging, data capture and biochemistries, and contemporary conceptual approaches now position this field to accelerate efforts in understanding and optimizing pregnancy, reaping the associated benefits for lifelong health.

To better explore and propose potential avenues to capitalize on new concepts and technologies, the Burroughs Wellcome Fund (BWF) supported a Pregnancy Think Tank meeting on November 19 to 20, 2019, in Research Triangle Park, North Carolina (See Box for description of BWF).

**Box:** About the Burroughs Wellcome Fund

The Burroughs Wellcome Fund (BWF) was established in the United States in 1955 on the legacy of Silas Burroughs and Henry Wellcome, two American-born colleagues who founded the Burroughs Wellcome Co. in London in the 1880s. The pharmaceutical company flourished through the 20th Century establishing entities around the globe. The Fund was the philanthropic arm of the North American branch of the pharmaceutical company. By the time of its creation, the corporation was run by the Wellcome Trust, a nonprofit entity created by the corporate shares of the global companies. In 1993, the Burroughs Wellcome Fund was the beneficiary of a $400 million gift from the Wellcome Trust. This endowment allowed BWF to become a completely independent foundation, with no direct ties to its founding company. The Reproductive Sciences have been a long-standing focus of BWF as being a critical yet underfunded, undervalued area of research. BWF history in this domain includes support for American Association of Obstetricians and Gynecologists Foundation (1997-2003), the Reproductive Scientist Development Program (1998 – present with the NIH), Frontiers in Reproduction, Marine Biological Laboratories Summer Course (1998 – present), and the Preterm Birth Research Consortium and the Preterm Birth Initiative (2007 – 2020) to initially support meetings and then fund discovery grants around preterm birth. This Think-Tank was the evolution of these initiatives to determine productive paths for the future discovery. BWF remains an independent private foundation whose mission is to advance the medical sciences by supporting research and other scientific and educational activities. BWF focus is on early stage biomedical scientists, particularly physician scientists, along with areas of investigation that are undervalued or underfunded by other organizations. George Hitchings, Ph.D., a Nobel laureate who spent most of his career with Burroughs Wellcome Co., served as BWF's president from 1974 until 1990, and his vision promoted the Fund's belief in the critical link of basic research and clinical applications in medicine. BWF is governed by a Board of Directors consisting of accomplished scientists and administrators, with many distinguished advisory committee members for its competitive award programs. These include HHMI Investigators, National Academies Members, and Nobel Laureates. For more information on the history of the Burroughs Wellcome Fund please see: https://www.bwfund.org/history.

The Think Tank meeting centered on generating novel ideas and stimulating new research opportunities for bolstering knowledge in the area of pregnancy and mechanisms underlying pregnancy complications. We were motivated by the joint biennial meetings that were convened by the BWF and the March of Dimes, starting in 2008 under the title “Preventing Prematurity: Establishing a Network for Innovation and Discovery,”^2^ and by recent initiatives such as the Magee-Womens Research Institute Summit. Considering the effect of those meetings on recent advances in basic, translational, clinical, and epidemiologic research and the advances that have emerged overall, the outlook for the field has expanded beyond the vision of BWF’s earlier Preterm Birth focus to include broader gestational conditions and their effect on lifelong maternal and infant health.

**Glossary of terms**

Altricial: born in an immature state requiring prolonged care

Artificial intelligence: the computation ability to process data, to learn from data analysis, and to apply and adapt to that learning to attain goals

Epigenetic: external modifications to DNA or RNA that lead to changes in gene expression without change to the nucleic acid sequence itself

Genome-wide association study (GWAS): a method commonly used in genetics to identify associations between common genetic variations occurring in populations with specific disease or traits

Genomics: the study of the structure, function, and evolution of genomes

Gene-regulatory elements: genome components that modulate transcriptional and posttranscriptional activity of genes. Examples include promoters and enhancers that are cis-acting DNA sequences, most often in noncoding regions of DNA that regulate transcription.

Hemotrophic: the transfer of blood-borne materials between the maternal and fetal circulations for nutrition of the fetus

Histotrophic: the use of extracellular material derived from the endometrium and the uterine glands that accumulates in the space between the maternal and fetal tissues for nutrition

Machine learning: the use of computational algorithms and statistical models that perform analyses without specific instructions

Mechanisms: the fundamental processes responsible for a specified action, phenotype, trait, or other natural phenomenon

Metabolome: the composition of the entire set of metabolites present within an organism or one of its compartments

Microbiome: microorganisms and their genes that populate a specific environment

Organoid: three-dimensional tissue constructs that are derived from stem cells in vitro that mimic the corresponding in vivo organ

Personalized medicine: healthcare individualized by a person’s unique genetic, environmental, and social characteristics

Phenome: the set of all phenotypes expressed by a biological compartment such as a cell, tissue, organ, or organism

Population medicine: clinical contributors to health that includes the important function of nonmedical stakeholders (education, social, business) to have a wider influence

Precocial: born in an advanced state capable of early survival with little support

Proteome: the composition of the entire set of proteins present within an organism or one of its compartments

Transcription factor: proteins that activate or repress genes by binding to DNA

Transcriptome: the composition of the entire set of RNA transcripts present within an organism or one of its compartments

Viviparous: giving rise to live birth from inside the body of the parent

Our event aimed to assess how convening experts from divergent biomedical disciplines, with many scientists from outside the field of pregnancy research, might foster productive and convergent thinking and generate novel ideas to stimulate innovative research projects. Together, 30 participants from the United States, Canada, and United Kingdom encompassed biological disciplines (Table 1). Our event was also designed to reshape the BWF strategy to accelerate discovery, encouraging proposals that incorporate new tools, analytic approaches, and scientific disciplines. This summary encapsulates the ideas and discussions organized by broad categories that span the contributions by experts in the fields (Table 1) and illuminate advances in ideas, technology, and talent to have the greatest potential: developmental genomics, physiology, and personalized and population medicine. We generated suggested questions and a call for scientific action, delineating next-generation challenges and opportunities in the science underlying pregnancy and human early development.

**Table 1:** Disciplines represented

Anatomy	Immunology
Bioengineering	Machine learning and artificial intelligence
Chronobiology	Microbiome and microbiology
Developmental biology	Obstetrics
Drug and device development	Pathology
Endocrinology	Pediatrics
Epidemiology	Personalized medicine
Evolutionary biology	Pharmacology
Genomics	Population genetics
Global health	Systems biology
Health policy	Vascular biology
Imaging sciences	

*Sadovsky. Pregnancy as a key window for discovery. Am J Obstet Gynecol 2020*.

## Developmental Genomics

**Figure 1:**
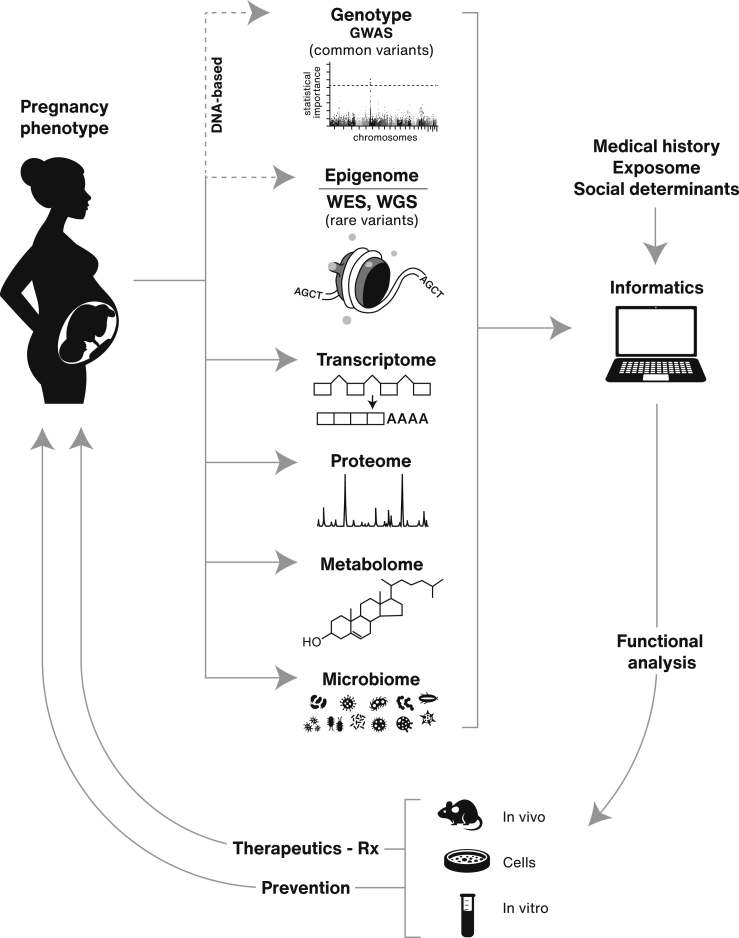
The cycle of discovery to strategies to prevent adverse pregnancy outcomes Human populations with new technologies and omics platforms, integrated with medical history, social determinants, and environmental data, provide unparalleled opportunities for precision and population medicine. *AGCT*, adenine, guanine, cytosine, thymine; *GWAS*, genome-wide association study; *Rx*, prescription; *WES*, whole exome sequencing; *WGS*, whole genome sequencing. *Sadovsky. Pregnancy as a key window for discovery. Am J Obstet Gynecol 2020.*

The field of developmental genomics applies state-of-the-art methodologies that strive to assist in bridging genotypes, physiology, and disease specifically to developmental processes from embryogenesis to parturition biology. From identification of genetic variants through sequencing or genome-wide association studies (GWAS) to defining nucleotide changes and investigating their downstream proteomic, metabolomic, and physiological consequences that may play an etiologic role in phenotypic consequences during pregnancy, numerous interrelated steps are logically aligned to chart the molecular maps underlying diseases^3^ (Figure 1). Pregnancy phenotypes are shaped by the maternal, fetal, and placental genomes and epigenomes, and therefore, the genomic studies of these phenotypes would benefit from an integrative approach in mother-infant pairs. Furthermore, these maps are not two-dimensional, but include regulatory elements that form three-dimensional DNA loop configurations to influence more than 1 proximal or distal gene.^4^ Notably, gene-regulatory elements comprise modular arrays of binding sites for transcription factors whose expression is context and time specific, changing across interacting maternal-fetal tissues and during gestational ages. These are further modified by environment-, diet-, or disease-induced epigenetic changes whose characterization and transmission are increasingly well understood.^5^^,^^6^ Generalizing these genomic processes across populations and ethnic groups can be accomplished with large databases and biospecimen banks with machine learning tools to probe them, guided, wherever possible, by biological reasoning and hypothesis formulation. To date, for most DNA sequence variants identified, relatively little mechanistic and actionable data have emerged. As associations now exist for preterm birth and birthweight, these should drive functional studies to translate the findings to improvements in clinical care.^3^^,^^7^ These functional studies should first indicate conclusive evidence for the actual genes responsible for the association because the nearest gene is not always the one relevant for the phenotype being investigated.^8^ Subsequent investigations to determine the mechanism of action of the causal polymorphism can be performed in vitro, in cell lines, and now in vivo using gene-editing technologies. These approaches determine when and how during pregnancy the variants modulate pregnancy outcomes.

### Incorporating evolution in pregnancy

By viewing the genesis of pregnancy through an evolutionary biology lens, significant insights have been gained, for example, into the signals required to maintain the long gestations that many mammals display. One recent elegant study by Griffith^9^ and colleagues found that by studying marsupial pregnancy the implantation reaction of eutherians (formerly “placentals”) derives from the attachment reaction in ancestral therian mammals (encompasses both eutherians and marsupials). In marsupials such as the opossum, this event leads to prompt parturition. The ability to shift from the inflammatory attachment reaction to a prolonged noninflammatory period in gestation is a central innovation in eutherian mammals that allowed an extended period of intimate placentation.

Although inferences based on evolutionary biology are powerful in mapping regulatory elements and traits at the organism-level, the rapid evolutionary changes in relevant organs, such as the placenta,^10^ may benefit from system-specific and well-thought-out machine learning approaches to link genomes to phenomes. Although the genomic roots of maternal-fetal tolerance and exchange are ancient, dating back to reptiles, fish, and velvet worms, at term only a small fraction of genes represent the core placental transcriptome that is conserved across mammalian or marsupial species.^11^ Moreover, eutherian and marsupial mammals have evolved imprinting (a way of generating monoallelic expression) as an additional mechanism to tune gene expression in a manner informed by parent of origin. It is possible that pharmacologically induced diseases in model organisms, such as the stimulation of preterm birth in mice, might not be as revealing as naturally arising variations in the timing of birth. In this instance, a mouse strain that evolved to spontaneously deliver its pups 3 days early might be more informative. This approach has been utilized in genetically inbred mouse strains to determine genetic contributors to gestational duration and also the coordination of gestational duration with rates of organ maturation.^12^^,^^13^ Importantly, the divergent strategies between species to optimize reproductive outcomes may in themselves prove informative for greater mechanistic insights into healthy and complicated pregnancies.

These challenges are further amplified by the general paucity in knowledge of maternal-fetal biology. Better tools such as next-generation sequencing, single-cell omics, and high-resolution imaging could bridge the divide between commonly used mouse and other gestational models and human pregnancy. The advent of single-cell sequencing of human placental cells, other cells at the maternal-fetal interface, and cells in the placenta of other model organisms may shed light on shared processes that might otherwise seem disparate. For example, single-cell transcriptomics from first-trimester placentas, maternal blood, and decidual cells identified regulatory interactions that are critical for immune adaptation and for placentation and diseases during pregnancy.^14^

## Gestational Physiology

It is now clear that the origins of many healthy developmental processes or diseases begin in the preconception period and in early pregnancy. For example, abnormal placental function may be rooted in defects of the trophoblastic shell, which encases the embryo within the maternal milieu.^15^ Research into cellular and organismal physiology has dramatically advanced in the past decade. GWAS and next-generation sequencing studies, single-cell omics, organoids, functional biochemical imaging, and other new technologies usher in ways to interrogate gestational physiology and major obstetrical syndromes.^14^^,^^16^, ^17^, ^18^, ^19^ During the critical phase of organogenesis across the first 8 to 10 weeks of human gestation, the endometrial glands supply secretions (commonly termed “uterine milk”) that support the developing embryo and are involved in placental development during the first trimester of pregnancy and spiral artery remodeling. A recently introduced hormone-responsive endometrial organoid model may shed light on this early histotrophic support to the embryo.^20^ Organoids, by generating a three-dimensional tissue construct in vitro from differentiating stem cells, may more faithfully reflect in vivo cellular function as related to the organ or tissue they model than traditional two-dimensional cultures of transformed or primary tissue culture cells.

Transcriptional profiling of the endometrial organoid cells indicated the strong similarity between organoids and the primary originating tissue. Furthermore, with exposure to pregnancy signals, endometrial organoids develop characteristics of early pregnancy and can be more faithfully used to model early gestational events. Although the precise composition of the endometrial gland secretions and their effects on placental development and organogenesis remain to be defined, the data suggest that premature onset of maternal blood flow into the placenta and transition from histotrophic to hemotrophic embryonic support is associated with early miscarriages. Could early gestational disorders reflect deficiencies in the new trophoblast-endometrial dialog and maladaptation to a 3-fold higher oxygen level or to new nutritional sources?

### Is pregnancy an immunologic paradox?

Despite being an allograft, in a healthy gestation, the maternal immune system does not reject the embryo, and pregnancy seems to be a precisely calibrated collaboration between the maternal and fetal needs, with the trophoblast forming a boundary between the 2 individual systems. Maternal T cells and decidual subsets of natural killer cells could potentially mediate allo-recognition—the recognition and response of maternal immune cells to placental trophoblast cells.^21^ Somatic fetal cells are separated by trophoblast from contact with the mother; thus, stricto sensu, “fetal rejection” cannot occur. Trophoblast organoids can now be used experimentally to investigate the interactions occurring between maternal decidual immune cells and the invading trophoblast, thus illuminating these critical transitions during the early stages of pregnancy.

### Reconsidering fetal growth

Beyond the first trimester, precise changes in fetal growth represent a complex physiological process that may not be well captured by simple growth curves. Importantly, fetal growth is saltatory, not steady.^22^^,^^23^ Deciphering normal fetal growth requires deep understanding of cell differentiation and the individual, temporal growth patterns in response to environmental influences. These may perturb organ growth trajectories, such as those expressed in growth plate biology, where cell differentiation defines bone elongation.

Natural selection may favor rapid intrauterine growth of the human brain during fetal development, driving birth to occur relatively early, either before the fetal head becomes larger than the pelvic outlet or owing to maternal metabolic constraints.^24^^,^^25^ Yet, such acceleration in birth timing leads to an altricial neonate in terms of neurodevelopment and the lack of early independence. The traits may have influenced endocrine signals between the fetoplacental unit and the mother, thus linking fetal maturation (especially brain development) to 1 of the triggers for parturition. For example, although the onset of term labor in most species is triggered by a steep prepartum drop in maternal serum progesterone concentration, the serum abundance of progesterone in women remains high throughout pregnancy.^26^ Nonetheless, loss of the progesterone block to labor seems to be a terminal event in the parturition cascade of all viviparous species studies to date.^27^ In human pregnancy, this is thought to be achieved by redundant mechanisms that interfere with or overcome progesterone receptor signaling in uterine cells, which may be influenced by systemic or local factors, such as signals from an overdistended lower uterine segment.

Pregnancy can be viewed as a physical, cardiovascular, immunologic, metabolic, physiological, and psychosocial stress test for the mother whose homeostatic systems are challenged by placental hormones to mostly favor uterine quiescence while providing the fetus with resources needed to complete its growth and developmental program. In the future, gestational age as a surrogate for the developmental age of the fetus may be replaced by a panel of other biomarkers more reflective of fetal readiness for being delivered. A mismatch between the production of placental hormones and the responses of maternal organ systems to those hormones may disrupt maternal homeostasis leading to disease. Therefore, a detailed chronological map of the placental-maternal endocrine landscape during human pregnancy under different circumstances and in different environments is a central goal that is now attainable through recent advances in omic technologies. Such a map would illuminate key processes in normal and adverse pregnancy outcomes and may identify clinical disease risk biomarkers.

## Integrated Personalized and Population Medicine

The implementation of personalized medicine and of population medicine have become high-priority frameworks, although they often seem to pull in opposite directions.^28^ Personalized or precision medicine aspires to use genomics and other data-driven methods such as medical history, environmental exposures, and social determinants for a given individual to improve diagnosis and optimize therapeutic decisions. These methods often blur the distinction between proven healthcare and research and can be costly. In contrast, population medicine seeks to structure, deliver, and make cost-effective high-value healthcare that improves the overall health of a given population. A key goal for medicine is to integrate the 2 approaches to achieve the best outcome for the largest number of patients. That integration is now a realistic goal and should be a high-priority framework for healthcare systems, patients, investigators, and funders. Capturing clinical evaluations along with deep phenotypes through data from electronic health records and integrating findings with genomics, social determinants, environmental insults, and health behaviors to develop personal health plans is a realistic goal.^29^^,^^30^ How do we exploit these advances to optimize pregnancy and lifelong health?

One addition to healthcare delivery today is that while we continue to emphasize the identification of specific diseases, there is a parallel proactive, holistic, and personalized approach to health, with participatory involvement from the individual that focuses more on wellness. Although throughout the 1900s, diseases were viewed as discrete autonomous entities, in the 2000s, many diseases are perceived in the context of baseline biological and genetic risks that interact with environmental factors. Medicine will always need disease-specific approaches for purposes such as organizing clinical management most effectively, controlling specific infectious diseases, or dealing with pandemic threats such as the coronavirus disease 2019 (COVID-19) that we are now facing. This additional focus complements the disease-specific approach by promoting health and impeding the progression of clinical disease. One avenue in realizing this health goal is an integrated systems approach to pregnancy.^31^^,^^32^ Network-based modeling and machine learning approaches that integrate diverse multiomic data from both mother and child and consider their possible interactions hold great promise. These approaches also enable the integration of other data sources that capture social, psychological, environmental, and other biological risk factors. These models may be informed by polygenic risk scores,^33^ nongenomic factors, or the integration of both. When stratified from low to high risk, they may be included in predictive algorithms that can indicate level of risk for a pregnant woman or her fetus for a particular disease. These models, especially when informed by an inclusive approach that incorporates environmental risk, may suggest individualized pathways to preventive medicine.

Improved pregnancy care should be paralleled by better approaches to care during infancy and childhood. Proactive, personalized models of health delivery with personal health plans should ideally promote patient engagement, anticipate and minimize risks, provide best care standards, and enable continuous adoption of new technologies as they are validated. The improved perinatal and neonatal care of recent decades that has incorporated new technologies as they emerge has enabled the survival of many premature and underweight babies who are at increased risk for a range of childhood and late-onset diseases. However, the challenge of preventing the diseases themselves remains. Preventive interventions deployed before birth, such as the administration of steroids or magnesium sulfate to mothers in preterm labor to reduce the risk of prematurity-related diseases, are relevant examples, and we hope that we may soon be able to address, using the new technologies described here, additional preventive approaches to the hazards to mothers and babies of perinatal inflammation; infectious agents; toxic, environmental, and social factors; and other contributors to perinatal injury.

### Leave no population behind

The development of personalized and population perinatal medicine should address the disparities in both information and outcomes across human populations.^34^ Predictive models are ultimately limited by available data, particularly in the context of racial and ethnic diversity. The overrepresentation of individuals of European ancestry in available genetic and environmental studies of pregnancy presents a risk that the deployment of personalized medicine approaches could perpetuate disparities in outcomes for underserved populations. The study of diverse populations (diverse in both genetic and environmental circumstances) will provide insights into biology and mechanisms. At the same time, pregnancy health is an area where disparities are prominent, and addressing them is a high priority if we are to improve pregnancy outcomes. We will need to take a population health perspective to fully embrace the promise of new technologies.

## Central Questions to Answer

The preceding discussions encompassing omics, physiology, and personalized and population medicine promote their considerations as powerful new tools and concepts that can ultimately improve health and prevent disease through knowledge of underlying mechanisms and understanding the effect of genetics, the environment, social determinants, medical history, and health behaviors in pregnancy. Strategies to implement measures for individuals and populations that take each of these factors into account can then be rationally developed. How can these areas be taken forward and moved into action? What will be the questions that will have to be answered and challenges to be overcome? Discussions from the Working Group developed the following lists.

### Development

• What are the mechanisms underlying the developmental origins of later-life health and disease? Are these transgenerational, and can the effects be mitigated or reversed?
• What are the evolutionary implications of the maternal and fetal genomes in shaping pregnancy phenotypes and transgenerational effects?
• Although animal models, particularly the mouse, are pertinent for some mechanistic studies in vivo, the extent to which they recapitulate various aspects of human pregnancy is unclear and may be limited. Can model choices be optimized for each clinical problem by harnessing comparative technologies to inform the precise extent of shared vs unique features of pregnancy between humans and other mammals?
• Can some animal models be further developed in the future, and should some models be replaced by organoids or other engineered ex vivo models?
• What are the biological mechanisms that define the gestational clock and the developmental clocks and how do they relate? How can these mechanisms be controlled?
• How can endometrial function be assessed and optimized before pregnancy?
• Can new standards be set for longitudinal studies of pregnancy, starting before conception or from the first trimester of pregnancy?
• How can the power of novel imaging technologies, alongside liquid biopsies of plasma and extracellular vesicles, to define time-dependent normal physiology and abnormal disease trajectories be harnessed?
• Can environmental vicissitudes be measured directly or with surrogates? Can they be integrated into the temporal trajectory of human pregnancy to explore the role of external factors in birth timing?

### Systems biology

• How can powerful, maternal-fetal multiomic approaches serve to redefine pregnancy health?
• What is the role of single-cell atlases, mass cytometry, and organoids in understanding pregnancy-related cell systems?
• Can a set of interacting organoid tissues capture a more complete system of gestational biology and be deployed to study development and even therapeutics?
• Should “reference” data sets of pregnant women and their offspring, which are large enough to include confounding factors, be developed and deidentified and be made publicly available for mining?
• Can better interaction with machine learning labs be stimulated, thus ensuring that cutting-edge technologies can be deployed rapidly and that investigations can better identify and classify novel variables?
• How do the evolutionary and demographic histories of different human populations modulate baseline risk for diseases of pregnancy?

### Diseases

• How can understanding of the effect of racial, ancestral, nutritional, and environmental differences in the pathobiology of perinatal diseases be accelerated?
• What initiates the onset of labor and the timing of birth, and what causes premature initiation of such signals?
• What truly defines the differential contribution of the placenta to major diseases of pregnancy: implantation failure and pregnancy loss, fetal growth restriction, preeclampsia, fetal death, and preterm birth?
• Can the power of modern technologies be harnessed to access the placenta and other intrauterine tissues in real time and throughout pregnancy and identify deviations toward disease even before they manifest clinically?
• Can “natural experiments,” such as surrogacy, help in understanding the immune basis of pregnancy loss and immunologic abnormalities of pregnancy?

### Clinical approaches and tools

• Can health hazards that can be safely and efficiently removed early in pregnancy be identified?
• What is the potential to collaborate with the wearable and sensor industry in the development of continuous monitoring systems during pregnancy and in alerting physicians about early deviations from “normal?”
• Can novel noninvasive tools be deployed for longitudinal assessment of pregnancy health?
• Can better involvement of the pharmaceutical industry be fostered to improve the safe deployment of new drugs or the repurposing of existing drugs during pregnancy?
• Can informatics tools, including artificial intelligence, be incorporated into drug discovery strategies?
• What will be the safest and most informative approaches to determine kinetics and efficacy of drugs in human pregnancy to prevent or treat pregnancy complications?
• What is the optimal path to enhance our engagement of ethicists in discussions about studies during pregnancy, particularly pregnancy monitors?

### Population health

• Is it possible to translate our basic science findings into actions that can produce measurable improvements of health at the population levels?
• What is the best strategy to examine naturally occurring variations in disease risk in pregnancy and the perinatal period in different populations to inform our scientific agendas?
• How can data arising from many levels—social, psychological, physiological, metabolic, and genomic—best be integrated into a real agenda for improved pregnancy health?
• How can newly arising population risks for poor pregnancy outcomes from infectious, environmental, social, or other changes as early as possible in their genesis be better prepared for?

## Summary: A Call for Scientific Action

**Figure 2:**
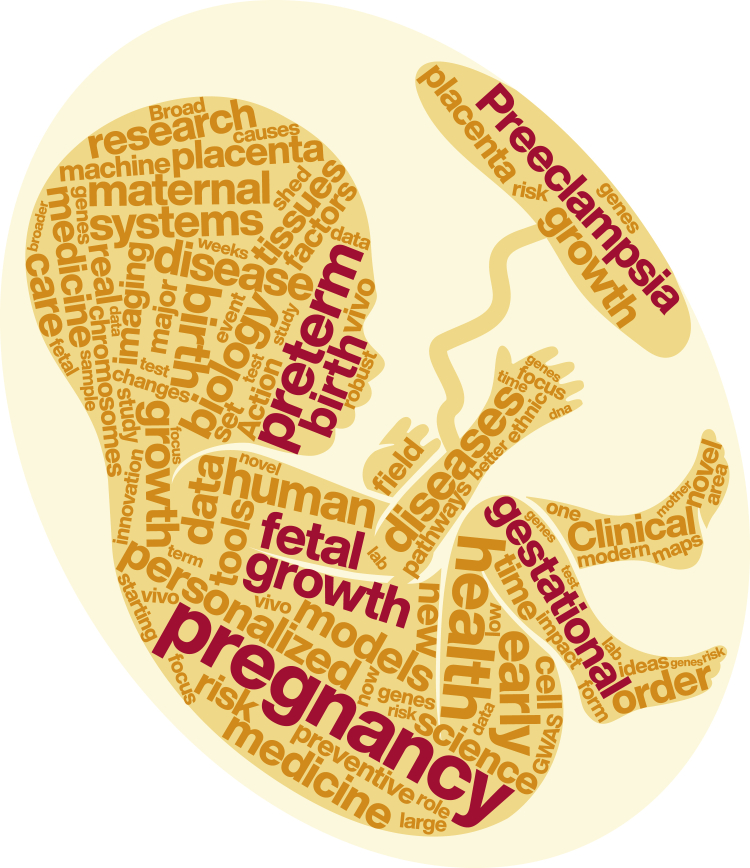
Word cloud of themes emerging from Pregnancy Think Tank discussions *Sadovsky. Pregnancy as a key window for discovery. Am J Obstet Gynecol 2020.*

While addressing diverse questions, Think Tank participants converged on several themes that cross biomedical disciplines and research trajectories (Figure 2). First and foremost, the transdisciplinary approaches weaved together by our diverse experts led to identification of key areas that need further exploration to advance and accelerate the solution to the identified complex challenges. The science underlying pregnancy and its translation to medicine is complicated and multidimensional, requiring the assembly of distinct expertise, working at many scientific levels and focusing on shared goals. Such assembly may pave the way to improve pregnancy health, personalized for the individual and applicable to the population and drawn up on the basis of all the data that can be collected and monitored for and by each patient. Disciplines not traditionally involved in the reproductive sciences, such as computer science, engineering, mathematics, and pharmacology, should be engaged at the study design phase to optimize the information gathered and to identify and further evaluate potentially actionable therapeutic targets.

There has been an impression that pregnancy research is not in vogue, creating an opportunity for new talented scholars to join the field. Greatly needed advances in the field will depend on the next generation of scholars. This should become a priority for the academic world. Furthermore, medical schools should place greater emphasis on training students to handle new multidimensional information and discuss it with patients, accommodating to the way care is poised to be delivered in the not-so-distant future.

Future research should include enough participants to span the diversity of human cohorts in terms of geography, racial and ethnic distributions, and social and health disparities. Broad studies must be introduced on the basis of ethical principles and regulatory compliance. To attain larger and more useful data sets, it may be time to include more heterogenous data and, thus, better reflect the “real world” of clinical practice, offering stronger conclusions. Bigger, more population-representative data sets will provide important insights that can be validated in more controlled settings. Modern technologies, for both data-gathering and data-analyzing, make this possible at a scale that was previously unachievable.

Research in the prepregnancy period and across the 9 months of pregnancy may benefit from the introduction of noninvasive, personalized sensors and monitors, alongside informative “liquid biopsies” for noninvasive pregnancy assessment. These will advance the application of mechanistic knowledge into human health and enrich disease-based and general precision medicine paradigms. Access to much-needed tissues from early pregnancy can be promoted by broader international collaborations. Furthermore, the psychosocial and economic environment in which pregnancy takes place cannot be ignored.

Overall, real progress in the understanding of pregnancy will take not only integrative science but also more time and, most importantly, more imagination. BWF and other funding agencies should find ways to promote innovation, not averting from “risk” in approaches. Academic departments at most universities should encourage and foster innovative, multidimensional, and transdisciplinary science and its application to integrative health and wellness.

## Supplementary Data

Supplemental File Burroughs Wellcome Fund Pregnancy Think Tank Working Group
