# Supplementary material for: Advancing human health in the decade ahead: pregnancy as a key window for discovery: A Burroughs Wellcome Fund Pregnancy Think Tank
Source: Am J Obstet Gynecol. 2020 Sep;223(3):312–21. doi: 10.1016/j.ajog.2020.06.031 (PMC7303037; doi:10.1016/j.ajog.2020.06.031)
Supplement: Supplemental File — Burroughs Wellcome Fund Pregnancy Think Tank Working Group [file mmc1.docx]

**Burroughs Wellcome Fund**

**Pregnancy Think-Tank Working Group**

**Nima Aghaeepour**

Stanford University

Stanford, CA

**Yaacov Barak**

Magee-Womens Research Institute

Department of OBGYN and Reproductive Sciences

University of Pittsburgh

Pittsburgh, PA

**J. Jay Boniface**

Sera Prognostics

Salt Lake City, UT

**Graham J. Burton**

University of Cambridge

Cambridge, United Kingdom

**John Anthony Capra**

Department of Biological Sciences

Vanderbilt University

Nashville, TN

**Sylvain Chemtob**

CHU Sainte-Justine

Montréal, QC, Canada

**Rachel M. Freathy**

University of Exeter

Exeter, United Kingdom

**Michael Greene**

Massachusetts General Hospital

Boston, MA

**John Hogenesch**

Cincinnati Children's Hospital Medical Center

Cincinnati, OH

**Michael Katz**

March of Dimes Foundation (emeritus)

Arlington, VA

Columbia University (emeritus)

New York, NY

Stanford University

Stanford, CA

University of Oxford

Oxford, United Kingdom

**Michelle Lampl**

Emory University

Atlanta, GA

**Anita Mahadevan-Jansen**

Vanderbilt University

Nashville, TN

**Sam Mesiano**

Department of Reproductive Biology

Case Western Reserve University

Department of Obstetrics and Gynecology,

University Hospitals of Cleveland, Cleveland OH, USA

**Ashley Moffett**

University of Cambridge

Cambridge, United Kingdom

**Louis J. Muglia**

Burroughs Wellcome Fund

Research Triangle Park, NC

**Jeffrey C. Murray**

Department of Pediatrics

University of Iowa

Iowa City, IA

**Marcelo A. Nobrega**

Department of Human Genetics

University of Chicago

Chicago, IL

**George Osol**

University of Vermont

Larner College of Medicine

Burlington, VT

**Nigel Paneth**

Departments of Epidemiology & Biostatistics and

Pediatrics & Human Development

College of Human Medicine

Michigan State University

East Lansing, MI

**Nathan D. Price**

Institute for Systems Biology (ISB)

Seattle, WA

**Jacques Ravel**

Institute for Genome Sciences, University of Maryland School of Medicine

Baltimore, MD

**Retz Reeves**

Islamorada Investment Management

Islamorada, FL

**Drucilla Roberts**

Massachusetts General Hospital

Boston, MA

**Yoel Sadovsky**

Magee-Womens Research Institute

Department of OBGYN and Reproductive Sciences

University of Pittsburgh

Pittsburgh, PA

**Rolly Simpson**

Burroughs Wellcome Fund

Research Triangle Park, NC

**John Sled**

Hospital for Sick Children

Toronto, Ontario, Canada

**Ralph Snyderman**

Duke Center for Presonalized Health Care

Duke University Medical Center

Durham, NC

**Derek E. Wildman**

Genomics Program

College of Public Health

University of South Florida

Tampa, FL

**Paul H. Wise**

Department of Pediatrics

Stanford University School of Medicine

Stanford University

Stanford, CA, USA

**Ge Zhang**

Division of Human Genetics

Cincinnati Children’s Hospital Medical Center

Department of Pediatrics

University of Cincinnati College of Medicine

Cincinnati, OH
